# Older Adults with Physical Frailty and Sarcopenia Show Increased Levels of Circulating Small Extracellular Vesicles with a Specific Mitochondrial Signature

**DOI:** 10.3390/cells9040973

**Published:** 2020-04-15

**Authors:** Anna Picca, Raffaella Beli, Riccardo Calvani, Hélio José Coelho-Júnior, Francesco Landi, Roberto Bernabei, Cecilia Bucci, Flora Guerra, Emanuele Marzetti

**Affiliations:** 1 Fondazione Policlinico Universitario “Agostino Gemelli” IRCCS, 00168 Rome, Italy; anna.picca@guest.policlinicogemelli.it (A.P.); francesco.landi@unicatt.it (F.L.); roberto.bernabei@unicatt.it (R.B.); emanuele.marzetti@policlinicogemelli.it (E.M.); 2 Department of Biological and Environmental Sciences and Technologies, Università del Salento, 73100 Lecce, Italy; raffaella.beli@unisalento.it (R.B.); cecilia.bucci@unisalento.it (C.B.); 3 Università Cattolica del Sacro Cuore, 00168 Rome, Italy; coelhojunior@hotmail.com.br

**Keywords:** aging, biomarkers, mitophagy, mitochondrial dynamics, mitochondrial quality control, mitochondrial-derived vesicles (MDVs), exosomes, mitochondrial-lysosomal axis

## Abstract

Mitochondrial dysfunction and systemic inflammation are major factors in the development of sarcopenia, but the molecular determinants linking the two mechanisms are only partially understood. The study of extracellular vesicle (EV) trafficking may provide insights into this relationship. Circulating small EVs (sEVs) from serum of 11 older adults with physical frailty and sarcopenia (PF&S) and 10 controls were purified and characterized. Protein levels of three tetraspanins (CD9, CD63, and CD81) and selected mitochondrial markers, including adenosine triphosphate 5A (ATP5A), mitochondrial cytochrome C oxidase subunit I (MTCOI), nicotinamide adenine dinucleotide reduced form (NADH):ubiquinone oxidoreductase subunit B8 (NDUFB8), NADH:ubiquinone oxidoreductase subunit S3 (NDUFS3), succinate dehydrogenase complex iron sulfur subunit B (SDHB), and ubiquinol-cytochrome C reductase core protein 2 (UQCRC2) were quantified by Western immunoblotting. Participants with PF&S showed higher levels of circulating sEVs relative to controls. Protein levels of CD9 and CD63 were lower in the sEV fraction of PF&S older adults, while CD81 was unvaried between groups. In addition, circulating sEVs from PF&S participants had lower amounts of ATP5A, NDUFS3, and SDHB. No signal was detected for MTCOI, NDUFB8, or UQCRC2 in either participant group. Our findings indicate that, in spite of increased sEV secretion, lower amounts of mitochondrial components are discarded through EV in older adults with PF&S. In-depth analysis of EV trafficking might open new venues for biomarker discovery and treatment development for PF&S.

## 1. Introduction 

Advancing age is associated with declining muscle mass, function, and strength, a condition referred to as sarcopenia which increases the risk of incurring negative health-related outcomes (e.g., disability, loss of independence, institutionalization, death) [1]. Hence, sarcopenia and its clinical correlates are major public health priorities. Physical activity, nutritional interventions, and multi-component programs have proven to be valuable strategies for managing sarcopenia [2,3,4]. Yet, no effective pharmacological treatments are currently available to prevent, delay, or treat sarcopenia, which is mostly due to the incomplete knowledge of the underlying pathophysiology [2]. To further complicate the matter, at the clinical level, sarcopenia shows remarkable overlap with frailty, a “multidimensional syndrome characterized by a decrease in physiological reserve and reduced resistance to stressors”, often envisioned as a pre-disability condition [5]. Hence, the two conditions have been merged into a new entity, referred to as physical frailty and sarcopenia (PF&S) [6].

Mitochondrial dysfunction and sterile inflammation are invoked among the pathogenic factors of PF&S [7,8]. Derangements at different levels of the mitochondrial quality control (MQC) machinery have been reported in older adults with PF&S [7]. However, whether and how cell-based alterations may spread at the systemic level and impact muscle homeostasis is presently unknown. 

One of the mechanisms by which cells communicate with each other involves a conserved delivery system based on the generation and release of extracellular vesicles (EVs) [9]. These vesicles transfer information between cells through several categories of cargo-enriched biomolecules (i.e., proteins, lipids, nucleic acids, and sugars), each of them selectively influencing different cellular domains [10]. This shuttle system also contributes to degradative pathways responsible for eliminating oxidized cell components, including mitochondria, by establishing inter-organelle contact sites [11]. In particular, in the setting of incomplete mitochondrial depolarization, cells may either delay autophagy to remove mildly damaged organelles or shift from mitophagy to the extrusion of mitochondrial components within EVs [12,13]. As such, the generation and release of mitochondrial-derived vesicles (MDVs) may represent a complement to MQC systems before whole-sale organelle is triggered [13,14].

Cell-free mitochondrial DNA (mtDNA) has been identified among the molecules released within exosomes that may act as damage-associated molecular patterns (DAMPs) [15]. One of the biological roles for these molecules is the activation of innate immunity through binding of their hypomethylated CpG motifs, resembling those of bacterial DNA, to membrane- or cytoplasmic-pattern recognition receptors (PRRs), including Toll-like receptor (TLR), nucleotide-binding oligomerization domain (NOD)-like receptor (NLR) [16], and cytosolic cyclic GMP-AMP synthase (cGAS)-stimulator of interferon genes (STING) DNA sensing system-mediated pathways [17]. However, mtDNA is not the only mitochondrial constituent that may be displaced via MDVs and trigger these responses. Recently, the extrusion of mitochondrial components other than mtDNA has been reported within small EVs (sEVs) purified from the serum of older adults with Parkinson’s disease (PD) [14]. However, whether and how this mechanism is in place in the setting of PF&S is unexplored. 

In the present study, we purified sEVs from older adults with and without PF&S, quantified their amount, and characterized their content for the presence of mitochondrial components. The identification of specific derangements in sEVs in PF&S may shed light on its pathophysiology as well as suggest new biomarkers and possible biological targets for drug development.

## 2. Materials and Methods

### 2.1. Participants

Older adults aged 70+ with and without PF&S were recruited among the participants of the “BIOmarkers associated with Sarcopenia and Physical frailty in EldeRly pErsons” (BIOSPHERE) study [18]. BIOSPHERE was designed to determine and validate a panel of PF&S biomarkers through multivariate statistical modeling of biomolecules pertaining to inflammation, redox homeostasis, amino acid metabolism, neuromuscular junction dysfunction, and muscle remodeling pathways [18,19,20]. 

The operational definition used in the “Sarcopenia and Physical fRailty IN older people: multi-componenT Treatment strategies” (SPRINTT) project [21,22] was applied to diagnose PF&S: (a) physical frailty, based on a summary score on the Short Physical Performance Battery (SPPB) [23] between 3 and 9, (b) low appendicular muscle mass (aLM), according to the cut-points proposed by the Foundation for the National Institutes of Health (FNIH) sarcopenia project [24], and (c) absence of mobility disability (i.e., inability to complete the 400-m walk test) [25]. The present investigation involved a convenience sample of 21 participants, 11 older adults with PF&S and 10 non-sarcopenic non-frail (non-PF&S) controls. Participants were randomly chosen from the cohort of the BIOSPHERE study [18], among those from whom serum was available for vesicle purification.

The study was approved by the Ethics Committee of the Università Cattolica del Sacro Cuore (Rome, Italy; protocol number BIOSPHERE: 8498/15) and all participants signed an informed consent prior to inclusion. Study procedures and criteria for participant selection were described thoroughly elsewhere [18].

### 2.2. Measurement of Appendicular Lean Mass by Dual X-Ray Absorptiometry

Appendicular lean mass was quantified through whole-body Dual X-Ray Absorptiometry (DXA) scans on a Hologic Discovery A densitometer (Hologic, Inc., Bedford, MA, USA) according to the manufacturer’s procedures. Criteria for low aLM were as follows: (a) aLM to body mass index (BMI) ratio (aLM_BMI_) < 0.789 in men and <0.512 in women, or (b) crude aLM < 19.75 kg in men and <15.02 kg in women [24].

### 2.3. Blood Sampling

Blood samples were collected in the morning by venipuncture of the median cubital vein after overnight fasting, using commercial collection tubes (BD Vacutainer^®^; Becton, Dickinson and Co., Franklin Lakes, NJ, USA). One blood tube was delivered to the centralized diagnostic laboratory of the Fondazione Policlinico Universitario “Agostino Gemelli” IRCCS (Rome, Italy) for standard blood biochemistry. The remaining tubes were processed for serum collection in the Biogerontology lab of the Università Cattolica del Sacro Cuore (Rome, Italy). Serum separation was obtained after 30 min of clotting at room temperature and subsequent centrifugation at 1000× *g* for 15 min at 4 °C. The upper clear fraction (serum) was collected in 0.5-mL aliquots and stored at −80 °C until analysis.

### 2.4. Small Extracellular Vesicles Isolation and Characterization

#### 2.4.1. Purification of Small Extracellular Vesicles by Differential Ultracentrifugation

Small EVs/exosomes were purified through differential centrifugation as previously described [14,26]. Briefly, serum samples were diluted with equal volumes of phosphate-buffered saline (PBS) to reduce fluid viscosity. Diluted samples were centrifuged at 2000× *g* at 4 °C for 30 min and pellets were discarded to remove cell contaminants. Subsequently, supernatants were centrifuged at 12,000× *g* at 4 °C for 45 min to remove apoptotic bodies, mitochondrial fragments, cell debris, and large vesicles (mean size > 200 nm). Supernatants were collected and ultracentrifuged at 110,000× *g* at 4 °C for 2 h. Pellets were recovered and resuspended in PBS, filtered through a 0.22-μm filter, and ultracentrifuged at 110,000× *g* at 4 °C for 70 min to eliminate contaminant proteins. Pellets enriched in purified sEVs were finally resuspended in 100 μL of PBS. To quantify sEVs, total protein concentration was measured using the Bradford assay [27]. 

#### 2.4.2. Western Immunoblot Analysis of Small Extracellular Vesicles

Western immunoblot analysis was performed to assess the purity of sEV isolation, to determine the type of sEVs on the basis of the expressed tetraspanins, and to characterize their protein cargo as previously described [14,28]. Briefly, equal amounts (1.25 μg) of sEV proteins were separated by sodium dodecyl sulphate polyacrylamide gel electrophoresis (SDS-PAGE) and subsequently electroblotted onto polyvinylidenefluoride (PVDF) Immobilon-P (Millipore, Burlington, MA, USA). Membranes were probed with primary antibodies against tetraspanins CD63 (1:200), CD9 (1:200), CD81 (1:200), a specific cocktail of antibodies (1:250) targeting mitochondrial markers (Table 1), flotilin (1:200), and heterogeneous nuclear ribonucleoprotein A1 (HNRNPA1; 1:1000). Technical specifications of primary antibodies used for Western immunoblotting are detailed in Supplementary Table S1.

The following day, membranes were incubated for 1 h at room temperature with anti-mouse peroxidase-conjugated secondary antibodies (1:2000) (Bio-Rad Laboratories, Inc., Hercules, CA, USA). Blots were visualized using the Clarity Max ECL Western Blotting Substrate (Bio-Rad Laboratories) and images were acquired by the ChemiDoc MP Imaging System and analyzed by Image Lab ^TM^ software version 6.0.1 (Bio-Rad Laboratories). Values of optical density (OD) units of each protein band immunodetected were normalized for the amount of sEV total proteins, as determined by the Bradford assay, and related to the control group, whose OD was set at 100%. 

#### 2.4.3. Analysis of Small Extracellular Vesicles by Scanning Electron Microscopy Imaging

Small EVs were fixed in a solution of 3.7% glutaraldehyde (Sigma–Aldrich, St. Luis, MO, USA) in PBS for 15 min, washed twice with PBS, and dehydrated through a series of ascending grades of ethanol (i.e., 40%, 60%, 80%, 96%–98%). Subsequently, samples were mounted on carbon adhesive stubs (Agar Scientifics, Stansted, UK) and left at room temperature for 24 h to obtain complete ethanol evaporation. Samples were gold-coated with a Balzers SCD 040 sputter coater (BAL–TEC AG, Balzers, Lichtenstein, Germany; thickness of gold layer: 40 nm) and analyzed at 132.21 K× magnification by a ZEISS EVO HD 15 Scanning Electron Microscope (Carl Zeiss Microscopy GmbH, Oberkochen, Germany) operating under high-vacuum at an accelerating voltage of 5 kV.

### 2.5. Statistical Analysis

Descriptive statistics were run on all data. Differences in demographic, anthropometric, and clinical parameters between PF&S and control participants were assessed via *t*-test statistics and χ^−2^ or Fisher exact tests, for continuous and categorical variables, respectively. All tests were two-sided, with statistical significance set at *p* < 0.05. Analyses were performed using the GraphPrism 5.03 software (GraphPad Software, Inc., San Diego, CA, USA).

## 3. Results

### 3.1. Characteristics of the Study Participants

The subset of participants included in the present study was representative of the whole BIOSPHERE cohort in terms of age, sex distribution, clinical characteristics, and body composition and functional parameters [8]. The main characteristics of study participants are presented in Table 2. Sex distribution, BMI, number of comorbid conditions and medications, total serum protein concentrations, and albumin levels did not differ between older adults with and without PF&S. PF&S participants tended to be older than controls, but the difference did not reach statistical significance. As per the selection criteria, SPPB scores and aLM either crude or adjusted by BMI were lower in older adults with PF&S relative to non-PF&S participants.

### 3.2. Characterization of Small Extracellular Vesicles from the Serum of Participants with and without Physical Frailty and Sarcopenia

#### 3.2.1. Verification of the Purity of Serum Small Extracellular Vesicles

**Figure 1:**
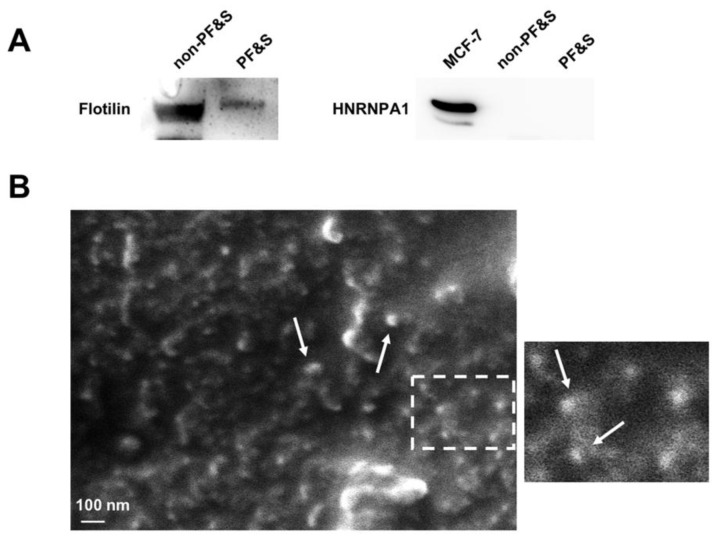
(**A**) Blots of the cytosolic protein flotilin and heterogeneous nuclear ribonucleoprotein A1 (HNRNPA1) as positive and negative markers respectively, in purified small extracellular vesicles (sEVs) obtained by serum ultracentrifugation from participants with physical frailty and sarcopenia (PF&S) and non-physically frail non-sarcopenic (non-PF&S) controls. The Michigan Cancer Foundation-7 (MCF-7) cell extract was used as the positive control for the anti-HNRNPA1 antibody. (**B**) Scanning electron micrographs of purified sEVs. The white-dashed box delimitates the area zoomed on the right. White arrows indicate some of the sEVs found in the observation field. Scale bar: 100 nm.

The purity of sEVs obtained by serum ultracentrifugation was ascertained according to the guidelines of the International Society of Extracellular Vesicles [29]. In particular, the presence of the cytosolic protein flotilin (positive control) and the absence of the non-sEV component HNRNPA1 (negative control) were verified (Figure 1A). The purified biospecimen was also analyzed by scanning electron microscopy (SEM) to confirm enrichment in sEVs. Small EVs appear in the scanning electron micrographs as objects of spherical shape and less than 100 nm in size (Figure 1B).

#### 3.2.2. Quantification of the Amount of Circulating Small Extracellular Vesicles

**Figure 2:**
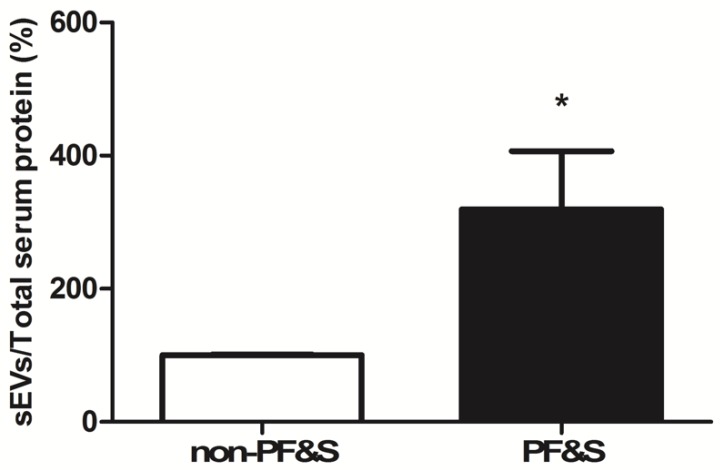
Serum levels of small extracellular vesicles (sEVs) in non-physically frail non-sarcopenic (non-PF&S) controls (n = 10) and participants with physical frailty and sarcopenia (PF&S; n = 11). Data were normalized for the amount of total serum proteins and are shown as percentage of the control group set at 100%. Bars represent mean values (±standard error of the mean). * *p* < 0.05 versus non-PF&S.

The total amount of sEVs purified from the serum of PF&S participants was significantly greater than in non-PF&S controls (*p* < 0.0001, Figure 2).

#### 3.2.3. Characterization of the Origin and Cargo of Small Extracellular Vesicles

**Figure 3:**
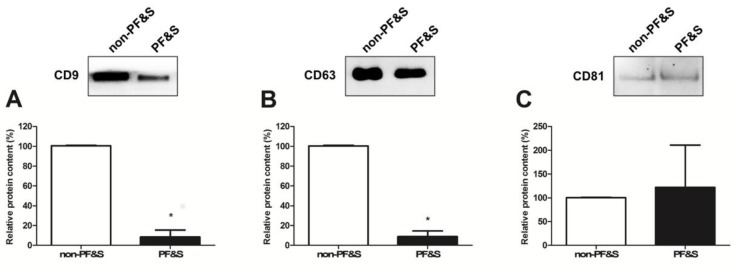
Protein expression of (**A**) CD9, (**B**) CD63, and (**C**) CD81 in purified small extracellular vesicles (sEVs) from non-physically frail non-sarcopenic (non-PF&S) controls (n = 10) and participants with physical frailty and sarcopenia (PF&S; n = 11). Data were normalized for the amount of sEV total proteins and are shown as percentage of the control group set at 100%. Bars represent mean values (±standard error of the mean). * *p* < 0.0001 versus non-PF&S.

Protein levels of the two tetraspanins, CD9 and CD63, were lower in participants with PF&S than in non-PF&S controls (Figure 3A,B), while CD81 content was unvaried between groups (Figure 3C).

**Figure 4:**
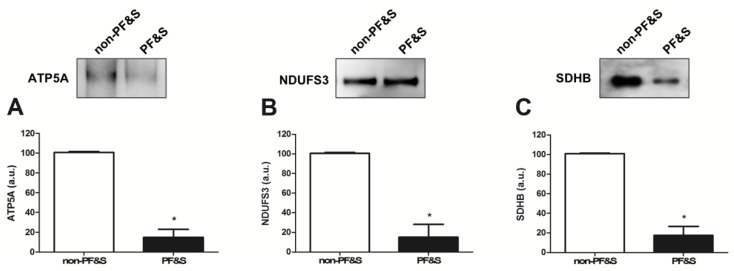
Protein expression of (**A**) adenosine triphosphate 5A (ATP5A), (**B**) nicotinamide adenine dinucleotide reduced form (NADH):ubiquinone oxidoreductase subunit S3 (NDUFS3), and (**C**) succinate dehydrogenase complex iron sulfur subunit (SDHB) in purified small extracellular vesicles (sEVs) from non-physically frail non-sarcopenic (non-PF&S) controls (n = 10) and participants with physical frailty and sarcopenia (PF&S; n = 11). Data were normalized for the amount of sEV total proteins and are shown as percentage of the control group set at 100%. Bars represent mean values (±standard error of the mean). * *p* < 0.0001 versus non-PF&S.

As for sEV cargo characterization, protein levels of adenosine triphosphate 5A (ATP5A; complex V), nicotinamide adenine dinucleotide reduced form (NADH):ubiquinone oxidoreductase subunit S3 (NDUFS3; complex I), and succinate dehydrogenase complex iron sulfur subunit B (SDHB; complex II) were lower in participants with PF&S than in non-PF&S controls (Figure 4A–C). No signal was detected for mitochondrial cytochrome C oxidase subunit I (MTCOI, complex IV), NADH:ubiquinone oxidoreductase subunit B8 (NDUFB8; complex I), or ubiquinol-cytochrome C reductase core protein 2 (UQCRC2; complex III) in either participant group.

## 4. Discussion

Among the factors involved in muscle degeneration associated with PF&S, mitochondrial dysfunction and the accrual of abnormal organelles have been indicated as relevant players [30]. However, the exact mechanisms underlying mitochondrial decay are not completely deciphered. 

Derangements in MQC processes have been reported in older adults with PF&S [7,31,32]. Nevertheless, alterations in sEV trafficking, which might contribute to MQC dyshomeostasis in muscle [33], have remained largely unexplored. To start filling this gap in knowledge, we purified sEVs from the serum of older adults with and without PF&S and, after ascertaining purity of the preparation, we determined the overall quantity of the mixed sEV population. Our results show a greater amount of sEVs in serum of PF&S participants compared with non-PF&S controls (Figure 2). The verification of the three tetraspanins, CD9, CD63, and CD81, in purified sEVs allowed these vesicles to be identified as a fraction of endosome-derived vesicles, referred to as exosomes, originating from the fusion of multivesicular bodies with the plasma membrane [28]. A lower protein expression of CD9 and CD63 was found in the exosome fraction purified from participants with PF&S (Figure 3), while levels of CD81 were comparable between groups. These observations are in keeping with the heterogenous composition of exosomes themselves, likely reflecting a different vesicle trafficking regulation [34]. Indeed, RAB27A, a guanosine triphosphatase (GTPase) that modulates exosome secretion, has been shown to regulate the secretion of CD63-positive exosomes, but not of those positive for CD9 [35]. Notably, exosomes derived by B-cells are characterized by the tetraspanin markers CD9 and CD81, while CD63 is absent [36]. A previous report by our group showed that RAB7A, a small GTPase and a master regulator of the late endocytic pathway, was able to modulate secretion of CD9- and CD81-positive exosomes [37]. The decreased expression of tetraspanin CD63 found in the present study may therefore be indicative of an altered late endocytic pathway [38], possibly suggesting disarrangements in late endocytic trafficking in PF&S.

The identification of mitochondrial components within the purified material allowed for classification of MDVs among sEVs. In particular, lower levels of the mitochondrial components ATP5A (complex V), NDUFS3 (complex I), and SDHB (complex II) were found in participants with PF&S (Figure 4). With the intent of preserving mitochondrial homeostasis, mitochondrial hyper-fission segregates severely damaged or unnecessary organelles [39,40] that are subsequently disposed via mitophagy [41]. However, mitochondrial-lysosomal crosstalk may dispose mildly oxidized mitochondria via MDV release [42]. Such a mechanism may therefore restore mitochondrial homeostasis before whole-sale organelle degradation is triggered [42]. Though, in the case of defective mitophagy or disruption of the mitochondrial-lysosomal axis, accrual of damaged mitochondria, misfolded proteins, and lipofuscin may occur as a result of inefficient cellular quality control [43]. Therefore, the increased sEV secretion in participants with PF&S (Figure 2) might reflect the cell’s attempt to extrude dysfunctional mitochondria. However, the reduced secretion of MDVs in the same participant group (Figure 4) may indicate that the MQC flux is impaired or that the damage to mitochondria is too severe to be disposed via MDVs. This idea is in keeping with previous reports by our group showing derangements in the expression of key proteins of the MQC machinery in old hip-fractured patients with sarcopenia [7,31].

The retrieval of mitochondrial components within sEVs is particularly relevant as it provides novel insights into the mechanisms of sterile inflammation, an age-associated inflammatory response mounted in the absence of infections [44]. This process is framed within the innate immune response and has been included as part of the ‘‘danger theory’’ of inflammation [45]. According to this view, misplaced noxious material from injured cells (i.e., damage-associated molecular patterns (DAMPs)) triggers caspase-1 activation and the secretion of pro-inflammatory cytokines [46]. The release of MDV content (e.g., mitochondrial proteins, mtDNA) can activate inflammatory pathways by interacting with several receptors/systems including TLRs, family pyrin domain-containing 3 (NLRP3) inflammasome, and cGAS-STING DNA sensing system [47].

Recently, we described the existence of a frailty “cytokinome” in older adults with PF&S defined by higher levels of P-selectin, C-reactive protein, and interferon-γ-induced protein 10, and lower levels of myeloperoxidase, interleukin 8, monocyte chemoattractant protein-1, macrophage inflammatory protein 1-α, and platelet-derived growth factor BB [8]. Pro-sarcopenic/pro-disability effects have traditionally been attributed to inflammation [48,49] as much as to dysfunction of anti-inflammatory pathways [49,50]. Furthermore, circulating MDVs have been identified in serum of older adults with PD and associated with a specific inflammatory profile [14]. However, the liaison among failing mitochondrial fidelity pathways, MDV secretion, and systemic inflammation may not be exclusive of neurodegeneration. Indeed, other conditions, such as HIV infection, a model of accelerated and accentuated aging [51], are characterized by pyroptotic bystander cell death and release of DAMPs that may trigger the same pathways as those identified in PD and inflamm-aging [52]. In addition, a massive release of DAMPs is acknowledged as a factor in the development of multiorgan failure in patients with severe injuries or during hemorrhagic shock [53]. Although the pathophysiology of multiple organ failure syndrome, neurodegeneration, and PF&S is heterogeneous, the release of mitochondrial DAMPs might be a converging mechanism shared by all of them. Should this assumption hold true, the scavenging of circulating mitochondrial DAMPs might represent a yet unexplored therapeutic option for the management of age-associated disarrangements, including PF&S. From this perspective, our findings are in line with the geroscience hypothesis, according to which the roots of most chronic diseases may reside in perturbations of a set of basic mechanisms (i.e., hallmarks of aging), including mitochondrial dysfunction [54]. 

Albeit presenting novel and promising findings, our work has limitations that need to be discussed. First of all, the cross-sectional design of the study precludes establishing cause–effect or temporal relationships between the analyzed pathways and PF&S pathophysiology. Also, although participants were carefully selected and thoroughly characterized, we cannot rule out the possibility that unknown comorbidities may have affected our results. In addition, our study provides an initial characterization of the heterogeneous population of circulating sEVs. Indeed, the analysis of the MDV cargo was limited to selected components/subunits of the mitochondrial electron transport chain. Hence, we cannot exclude that the analysis of other biomolecules, including mtDNA, that may be transported along the same road could provide additional insights into the relationship between sEV trafficking and PF&S. Finally, a deeper characterization of sEVs for their structure and content by means of transmission electron microscopy analysis is needed to confirm and expand our findings as well as to gain further information into the dynamic regulation of vesicle trafficking in PF&S.

## Figures and Tables

**Table 1:** Mitochondrial components and related electron transport chain complexes assayed in purified small extracellular vesicles by Western immunoblotting.

Mitochondrial Marker	ETC Complex
ATP5A	V
MTCOI	IV
NDUFB8	I
NDUFS3	I
SDHB	II
UQCRC2	III

Abbreviations: ATP5A, adenosine triphosphate 5A; ETC, electron transport chain; MTCOI, mitochondrial cytochrome C oxidase subunit I; NDUFB8, nicotinamide adenine dinucleotide reduced form (NADH):ubiquinone oxidoreductase subunit B8; NDUFS3, NADH:ubiquinone oxidoreductase subunit S3; SDHB, succinate dehydrogenase complex iron sulfur subunit B; UQCRC2, ubiquinol-cytochrome C reductase core protein 2.

**Table 2:** Participant characteristics according to the presence of physical frailty and sarcopenia.

Characteristic	Non-PF&S (n = 10)	PF&S (n = 11)	*p*-Value
Age (years), mean ± SD	73.9 ± 2.7	77.7 ± 5.4	0.0557
Gender (female), n (%)	5 (50)	8 (73)	0.5344
BMI (kg/m^2^), mean ± SD	28.1 ± 2.8	30.3 ± 4.3	0.1891
SPPB summary score, mean ± SD	12.0 ± 1.0	7.0 ± 0.3	<0.0001
aLM (kg), mean ± SD	20.21 ± 4.10	15.84 ± 3.63	0.0390
aLM_BMI_, mean ± SD	0.81 ± 0.32	0.51 ± 0.11	0.0118
Albumin (g/L), mean ± SD	45.4 ± 12.7	39.8 ± 1.2	0.1536
Total serum protein concentration (g/L), mean ± SD	71.8 ± 4.6	75.5 ± 3.1	0.0914
Number of diseases ^¥^, mean	3.2 ± 1.6	3.1 ± 1.2	0.8647
Number of medications ^#^, mean ± SD	2.9 ± 1.6	3.2 ± 1.8	0.7061

Abbreviations: aLM, appendicular lean mass; aLM_BMI_, aLM adjusted by body mass index (BMI); non-PF&S, non-physically frail non-sarcopenic; PF&S: physical frailty & sarcopenia; SD: standard deviation; SPPB: short physical performance battery. ^¥^ includes hypertension, coronary artery disease, prior stroke, peripheral vascular disease, diabetes, chronic obstructive pulmonary disease, and osteoarthritis. ^#^ includes prescription and over-the-counter drugs

## Supplementary Materials

The following is available online at https://www.mdpi.com/2073-4409/9/4/973/s1: Table S1: Technical specifications of the primary antibodies used for Western immunoblotting.
