# Supplementary material for: Older Adults with Physical Frailty and Sarcopenia Show Increased Levels of Circulating Small Extracellular Vesicles with a Specific Mitochondrial Signature
Source: Cells. 2020 Apr 15;9(4):973. doi: 10.3390/cells9040973 (PMC7227017; doi:10.3390/cells9040973)
Supplement: Supplementary file 1 [file cells-09-00973-s001.pdf]

Table S1. Technical specifications of the primary antibodies used for Western immunoblotting.

Antibody	Manufacturer and Catalog Number	Type	Species	Dilution	Detected Band MW (kDa)
ATP5A (complex V)<br>MTCOI (complex IV)<br>NDUFB8 (complex I)<br>SDHB (complex II)<br>UQCRC2 (complex III)	Abcam (Cambridge, MA, USA) ab1104413	Monoclonal	Mouse	1:250	55<br>40<br>20<br>30<br>48
CD9	Santa Cruz Biotechnology (Santa Cruz, CA, USA) (sc-13118)	Monoclonal	Mouse	1:200	25
CD63	Santa Cruz Biotechnology (sc-5275)	Monoclonal	Mouse	1:200	26
CD81	Santa Cruz Biotechnology (sc-166020)	Monoclonal	Mouse	1:200	25
NDUFS3 (complex I)	Santa Cruz Biotechnology (sc-374283)	Monoclonal	Mouse	1:200	25
Flotilin	Santa Cruz Biotechnology (sc-74566)	Monoclonal	Mouse	1:200	48
HNRNPA1	Santa Cruz Biotechnology (sc-32301)	Monoclonal	Mouse	1:1000	36

Abbreviations: ATP5A, adenosine triphosphate 5A; MTCOI, mitochondrial cytochrome C oxidase subunit I; HNRNPA1, heterogeneous nuclear ribonucleoprotein A1; MW, molecular weight; NDUFB8, nicotinamide adenine dinucleotide reduced form (NADH):ubiquinone oxidoreductase subunit B8; NDUFS3, NADH:ubiquinone oxidoreductase subunit S3; SDHB, succinate dehydrogenase complex iron sulfur subunit B; UQCRC2, ubiquinol-cytochrome C reductase core protein 2.
